# The Role of Wastewater Treatment Plants in Dissemination of Antibiotic Resistance: Source, Measurement, Removal and Risk Assessment

**DOI:** 10.3390/antibiotics13070668

**Published:** 2024-07-18

**Authors:** Kezia Drane, Madoc Sheehan, Anna Whelan, Ellen Ariel, Robert Kinobe

**Affiliations:** 1 College of Public Health Medical and Veterinary Sciences, James Cook University, Townsville, QLD 4811, Australia; ellen.ariel@jcu.edu.au; 2 College of Science, Technology, and Engineering, James Cook University, Townsville, QLD 4811, Australia; madoc.sheehan@jcu.edu.au; 3 Townsville Water and Waste, Wastewater Operations, Townsville, QLD 4810, Australia; aw3@townsville.qld.gov.au

**Keywords:** mobile genetic elements, horizontal gene transfer, membrane bioreactor, antimicrobial resistance, sewage processing, environmental pollution, One Health

## Abstract

Antibiotic Resistance Genes (ARGs) are contaminants of emerging concern with marked potential to impact public and environmental health. This review focusses on factors that influence the presence, abundance, and dissemination of ARGs within Wastewater Treatment Plants (WWTPs) and associated effluents. Antibiotic-Resistant Bacteria (ARB) and ARGs have been detected in the influent and the effluent of WWTPs worldwide. Different levels of wastewater treatment (primary, secondary, and tertiary) show different degrees of removal efficiency of ARGs, with further differences being observed when ARGs are captured as intracellular or extracellular forms. Furthermore, routinely used molecular methodologies such as quantitative polymerase chain reaction or whole genome sequencing may also vary in resistome identification and in quantifying ARG removal efficiencies from WWTP effluents. Additionally, we provide an overview of the One Health risk assessment framework, as well as future strategies on how WWTPs can be assessed for environmental and public health impact.

## 1. Introduction

Chemical pollutants that are collectively termed Contaminants of Emerging Concern (CEC) are classified as non-traditional unmeasured pollutants, distinguished by characteristics such as potentially high environmental risk at very low concentrations [1]. Wastewater Treatment Plants (WWTPs) are traditionally associated with disseminating CEC into receiving environments, specifically fresh and marine aquatic ecosystems [2]. Antibiotics, Antibiotic-Resistant Bacteria (ARB), and Antibiotic Resistance Genes (ARGs) have recently been identified as CEC from WWTPs [1], and these pose enormous risk to both public and environmental health. Interest is growing in the need to identify accurate assessment scaffolds to predict the risk of ARB and ARGs released from WWTPs to public health, animals, and the environment. There is concern in the scientific community, however, that the current state of knowledge on the dissemination of ARB and ARGs from WWTPs cannot be accurately applied to predict the risk to One Health.

### Antibiotic Resistance in Wastewater

The identifying trait of an antibiotic is to kill a bacterial cell or disable its ability to replicate. This is a natural process that organisms have used for millions of years to evolve in hostile environments and compete for resources. Antibiotic resistance (which is a subset of antimicrobial resistance) has already been identified as contributing to tens of thousands of human deaths per year in Europe and the USA alone [3]. To date, there are over 100 clinically used antibiotics, which are either developed from microbial isolates (e.g., erythromycin from *Saccharopolyspora erythraea*), or made synthetically (e.g., ciprofloxacin) [4]. Alternatively, some antimicrobial compounds are synthetic derivatives of naturally produced antibiotics (e.g., amoxicillin is a semi-synthetic derivative of penicillin). Each of these naturally occurring or synthetic antibiotics generally fit within specific classes including penicillins, macrolides, cephalosporins, fluoroquinolones, carbapenems, tetracyclines, sulphonamides, aminoglycosides, phenicols, lincosamides, glycopeptides, oxazolidinones and rifampicin. For each of these antimicrobial classes, general mechanisms of action and resistance have been reviewed previously [5]. It is known that genes encoding for antimicrobial resistance are continuously evolving and are significantly more varied than the number of existing antimicrobial compounds. The extensive variability and overlap in mechanisms of resistance has led to marked interest in characterizing the risk factors for resistance against each class of compounds.

The majority of currently available scaffolds for assessing risks for the highly diverse antimicrobial resistance pollution from WWTPs have suggested that the quantity and quality of antibiotics, ARB, ARGs as well as non-antibiotic determinants of resistance should be established from the sources of pollution for a proper risk evaluation in the One Health context [6,7]. Additionally, the available risk assessments indicate that vastly different microbiomes and resistomes exist within different WWTPs and their receiving environments, possibly due to a multitude of factors including marked diversity in influent sources and wastewater treatment processes, suggesting a need for an individualized risk assessment for each WWTP [6,7]. The most common sources of antibiotics and ARB in urban environments include hospitals and animal health clinics, industrial agriculture production facilities, and domestic sewage that is often laden with antibiotics from general medical use within the local population [8]. Most antibiotics are poorly biotransformed after consumption, and in some cases, up to 90% of the dose can be expelled un-metabolized or as active conjugates through urine and faeces [7]. WWTPs provide an opportunity to monitor and contain antibiotic resistance as all major sources and drivers of resistance tend to converge in urban WWTPs. As such, urban WWTPs are often considered hotspots for antibiotics and ARB and have been the subject of much investigation [9]. The processes involved in some forms of wastewater treatment have been observed to lead to significant variations in the microbial composition of WWTP effluents, and these rearrangements are reflected in the composition of antibiotic resistance harboured by the changing microbial communities [10,11,12,13]. Furthermore, in most jurisdictions globally, the dissemination of microbial contaminants within the effluent is generally not controlled or regulated, and no currently implemented wastewater treatment options are capable of total elimination of ARGs in effluents [14,15]. As a result, therefore, wastewater treatment facilities are still considered hot spots for potential amplification and dissemination of antibiotic resistance [16], despite a large reduction in the abundance of ARB and ARGs in some tertiary forms of wastewater treatment. Even relatively small abundances of ARGs, ARB, or antibiotics released into receiving environments can provide both the means and opportunities for the perpetuation of antibiotic resistance within environmental bacterial communities [15].

This review briefly outlines factors that influence the presence and relative abundance of ARGs in effluents that are discharged from WWTPs. More specifically, elements that lead to the acquisition of ARGs by susceptible bacteria, as well as effects of influent quality and intra-plant secondary and tertiary wastewater treatment processes, are described. In addition, a framework for assessing the risk to the environment from dissemination of ARGs associated with WWTPs is highlighted.

## 2. Approaches for Identifying Antimicrobial Resistance Factors in WWTPs and the Environment

### 2.1. Identifying ARB and ARGs

Vastly different methods are used to identify and quantify antibiotic resistance within WWTPs, WWTP-effluents and receiving environments. Most risk assessment scaffolds agree that testing for antibiotic and antibiotic resistance pollution within WWTPs should be standardised [17,18]. Consistency in methods for the identification of ARB, ARGs, and antibiotics is necessary, so that quantitative or qualitative values may be accurately compared between different WWTPs and associated receiving environments.

Although bacterial culture and specific antibiotic sensitivity testing is less common, some studies have used this technique to quantify ARB and putative corresponding intracellular ARGs (iARGs) in wastewater [19,20,21]. Bacterial culture-based approaches can be helpful when assessing wastewater for specific bacterial species/strain and especially pathogenic strains and their associated resistance mechanisms. These approaches provide the added benefit of observing the effects of specific stressors under controlled conditions, as well as identification of functional ARGs and virulence factors. Culture methods have also been used in tandem with gene sequencing techniques, which can detect ARGs harboured by specific bacteria [22,23]. However, cultivation-based approaches limit whole resistome and microbiome assessments, as only a small fraction of environmental bacteria are capable of growth under known laboratory conditions [24]. Additionally, bacterial culture does not provide information on the identification or quantification of environmental, extracellular ARGs (eARGs). This, therefore, may represent a selective bias as opportunities to characterise the full scale of ARGs and the associated microbiome within WWTP samples are lost.

Currently, the bulk of knowledge on evaluation of scope and frequency of antibiotic resistance in WWTPs has largely relied on the use of quantitative Polymerase Chain Reaction (qPCR) and Whole Genome Sequencing (WGS) approaches. With qPCR, specified ARGs can be detected and quantified in environmental samples, and recent advances in high-throughput qPCR arrays have enabled the characterisation of hundreds of ARGs simultaneously [25,26,27,28,29]. The use of the qPCR method for the detection of ARGs from wastewater also provides the opportunity for high-resolution quantification of specified sequences of ARGs within an array. However, the selectivity of primers for qPCR assays limits the identification of ARGs because the breadth of a resistome often encompasses more ARG sequences than could be feasibly tested via qPCR. This may provide further bias to the characterisation of WWTP resistomes. This bias is notable in this scientific field, with observable reliance on a few specific genes in several publications, and these include ARGs encoding for resistance against beta-lactams (blaCTX-M, blaTEM, and blaSHV), tetracyclines (TETM, TETO, TETQ, and TETW), sulphonamides (SULI and SULII), and class 1 integron-integrase (INTI1), which indicate the presence of mobile genetic elements (MGEs) [30,31,32,33,34].

More recently, WGS has been used extensively for the identification of ARGs in WWTPs and environmental samples [17,35,36,37]. This approach is helpful for not only identifying known ARGs [35,36,37], but also for its potential to identify novel ARGs [38], while simultaneously identifying the microbiome within WWTPs [35,37]. WGS methods are expensive, require a significant amount of bioinformatic skills, and gene identification is heavily reliant on reference databases and bioinformatic platforms and software to process raw reads, to generate interpretable data [39,40,41]. Furthermore, the quantification of gene expression by WGS is only determined as relative abundance because it is limited by gene characteristics such as nucleotide sequence length and abundance within the profiled resistome, and both qPCR and WGS approaches do not provide immediate information on the functional capability of identified ARGs. To gain an insight into whether ARGs identified by WGS have a functional capability, WGS is often coupled with advanced transcriptomic and proteomic analyses. A benefit of WGS is the volume of data generated, and it can facilitate the identification of different ARG variants as well as novel ARGs and bacterial chromosome sequences [42]. Given the strengths and limitations of each of the approaches used to characterise ARB and ARGs in wastewater and WWTPs, a clear set of guidelines for each of these techniques and consistent reporting of such details would enable better data comparisons across scientific fields and facilitate the acquisition of data that is necessary to accurately predict risks to the environment from sources of antibiotic resistance pollution. This argument has also been advanced by others in this field [17].

### 2.2. Identifying Antibiotics

Assessing WWTPs and associated receiving environments for antibiotics as selective agents for antimicrobial resistance is inconsistent across a number of studies. While some studies have tested for, identified, and quantified specific antibiotics of interest in wastewater and WWTPs [43,44], in other studies, this has not been done at all [30,45]. The presence and abundance of antibiotics provides an opportunity for ARGs to spread horizontally and/or vertically in bacterial communities, creating novel ARB. Analysing these compounds and their effective concentrations in conjunction with ARB and ARGs can markedly improve ecological risk assessments [17,43].

The presence and concentrations of antimicrobial compounds in wastewater and WWTPs have been used as indicators for conditions under which selection pressure would lead to the development and spread of ARB. To evaluate the efficacy and clinical applicability of antibiotics in laboratory settings, the concept of Minimum Inhibitory Concentrations (MICs) that inhibit bacterial growth in culture has been established. MICs are commonly applied to determine resistance or susceptibility to specific antibiotics [46,47]. By contrast, there is also increased interest in determining effects of exposing bacteria to antibiotic concentrations below respective MIC values [48,49]. Lower concentrations (<MICs) of antibacterial compounds have multiple effects, including the stimulation of Horizontal Gene Transfer (HGT) of ARGs through conjugation [50,51] and changing the transcription profiles of virulence factors for some pathogenic bacteria [52]. Furthermore, low levels of antibiotics have also been linked to the activation of the SOS response signaling and uptake of extracellular DNA (eDNA) [53], and, changing gene regulation through quorum sensing to increase bacterial cell variability [42,54]. Given that myriad effects are attributed to antibiotics at sub-lethal concentrations for bacteria, recent studies have focused on determining Predicted No-Effect Concentrations (PNECs), as concentrations at which different types of antibiotics may be present but have no effect on the microbiome [18,48,55]. PNECs are expected to become crucial in the monitoring and control of antibiotic pollution, and this would streamline risk assessment approaches with measures that are used for other CECs. It is important to note, however, that the PNEC of an antibiotic is likely to vary with the bacterial composition against which an antibiotic is tested, and the laboratory culture conditions for this validation process may further bias this scientific field. Furthermore, the assessment of antibiotics alone does not provide context for the risk of transmission of ARGs within the microbiome in receiving environments, and as such should not be the sole basis for the assessment of antibiotic resistance and the risk it may pose to an environment. There are other challenges to accurately reporting levels of antibiotics in WWTPs and environmental samples. For instance, the sorption of antibiotics to sediments in aquatic ecosystems, and marked variations in limits of detection or quantification of antibiotics by different testing methods will limit accurate assessment [56]. Antibiotics are not the only known selective agent for antimicrobial resistance, and, in effect, antibiotics may act synergistically or additively with heavy metals, biocides, pharmaceuticals and other emerging organic contaminants in wastewater to increase the abundance of specific ARB [57,58]. It is still highly debated whether this is due to selection and induction of the expression of co-resistance genes, enhanced adaption of resistance gene cassettes, or altered physiological/biochemical properties that upregulate ARG expression [56]. Nevertheless, the availability of known selective agents other than antibiotics should be considered when assessing the risk of antibiotic resistance pollution in WWTP-receiving environments.

## 3. Effects of Wastewater Treatment Processes on ARGs Released from WWTPs

Antibiotics contained within influent sources can enter WWTPs and allow resistant bacterial populations to propagate with less competition from susceptible bacteria strains. This is evidenced by positive correlations between specific antibiotics in the influent and their cognate ARGs in WWTPs [59,60]. For example, antibiotics of the macrolide class (clarithromycin and azithromycin) correlated positively with a macrolide resistance gene (MACB) in wastewater [59], and an increase in the total abundance of ARGs identified by qPCR in the activated sludge was observed after the addition of tetracycline [60]. Furthermore, it has been noted that up to 50% of antibiotic concentrations in influents are retained throughout the treatment process and the retained antibiotics are detectable in WWTP effluents [61]. Despite these specific examples, drawing broad and generalizable conclusions on the link between the presence of antibiotics and their associated ARGs remains difficult. For example, a positive correlation was observed between the concentration of tetracyclines and the number of cultured bacteria resistant to tetracycline, but there was no significant correlation between seven other antibiotics and their related culturable ARB [62]. Additionally, Xu et al. (2015) observed no link between the presence of sulfonamides and common sulfonamide resistance genes (SUL1 and SUL2) identified by qPCR [63]. By contrast, a negative correlation was observed when the abundance of a quinolone resistance gene (QNRC) decreased as the concentrations of enrofloxacin (a quinolone) increased in the effluent from a WWTP [63]. This phenomenon suggests that the relative abundance of an antibiotic may affect the presence but not necessarily the abundance of genes associated with resistance to that specific antibiotic [7]. Comparisons between different studies and identification of trends in the literature surrounding the interaction of ARB, ARGs, and antibiotics is also made difficult by the inconsistency in methods used to investigate these correlations. In addition, treatment type and the operational processes of individual WWTPs are also influential factors in reducing total bacteria and, by extension, the abundance of ARB and ARGs in effluents [64]. In this review, emphasis is placed on the impact of wastewater treatment processes on ARGs in the effluent (liquid phase) released from WWTPs (Appendix A Table A1). At most modern WWTPs, many different technological approaches are used in processing wastewater in what is classified as primary, secondary, and tertiary treatment [7].

### 3.1. Primary Wastewater Treatment

Primary treatment generally involves physical screening and removal of large solid, particulate matter. Despite there being limited literature on the effect of primary treatment on ARGs, the available evidence seems to indicate that primary physical treatment does not significantly remove ARGs, as noted for TETA, TETO, TETW, SULI, SULII, blaCTX-M, blaTEM and blaSHV following identification by qPCR [65,66]. It should be noted, however, that some forms of eARGs and eDNA can generally sorb to solid particles; speculation exists that a limited amount of such DNA molecules could still be removed from influents into WWTPs during the primary treatment phase [64].

### 3.2. Secondary Wastewater Treatment

Secondary wastewater treatment is usually characterised by a biological treatment step, where specific conditions are applied to the wastewater to explicitly cultivate specific bacterial species to break down biological material and nutrients. The biological step in secondary treatment may include an aerobic process and/or an anaerobic process as well as anoxic and/or oxic processes. The composition of bacterial communities throughout a WWTP are intricate [67], and the artificially changed oxygen and nitrogen availabilities throughout these processes drastically rearranges the wastewater bacterial community. Many studies, utilising both WGS and qPCR methods, have noted that this process results in a reduction in ARBs and their iARGs [15,59,65,68]. Similarly, in studies utilising both WGS and bacterial culture paired with qPCR, the total abundance of ARGs was found to decrease throughout WWTPs as the abundance of bacteria declined [37,48,69,70]. Therefore, this reduction is likely because ARGs follow a similar distribution to that of total bacteria during wastewater treatment since the major removal of bacteria via sludge separation occurs after the anaerobic and aerobic processing [68]. By contrast, there are instances where qPCR-based methods identified ARB and ARGs increasing in relative abundance when compared to total bacterial and gene loads [29,61]. To date, there has been very limited focus on the link between the composition and distribution of specific bacterial hosts of ARGs (i.e., ARB) within critical sections of WWTPs and whether this distribution is similar in both the influent and the effluent. Using qPCR, emulsion paired isolation, and concatenation PCR to detect bacterial hosts of ARGs, Hultman et al. (2018) observed that the distribution of four ARGs and the bacterial cells, which harboured these genes, varied between the WWTP influent and effluent [29]. A subsequent study found that the composition of the WWTP microbiome changed markedly throughout the WWTP and that bacterial hosts of ARGs and MGEs monitored by qPCR also changed throughout the plant [15]. These observations are likely to be attributed to the high incidence of HGT and sharing of ARGs on MGEs within WWTPs, and this speculation was based on identification by qPCR of many ARGs, including INTI1, blaTEM, blaOXA-A, blaSHV, blaCTX-M, SULI, SULII, and QNRS [68].

Further evidence of HGT of ARGs in WWTPs can be seen on an individual gene level. For example, the removal efficiency of a specific sulfonamide resistance gene (SULI) in biological treatment was investigated in four separate studies [71,72,73,74]. All four studies utilised the same qPCR primer and observed varied changes in the absolute abundance of other cumulatively measured ARGs as well as SULI. No difference in the relative abundance of SULI to bacterial load after biological treatment or a significant increase in the relative abundance of SULI after treatment was observed [71,73], while a decrease in the relative abundance of SULI was observed after treatment [72]. This inconsistency in specific gene removal by secondary treatment processes may be attributed to two plausible factors. First, ARGs such as SULI may be conserved within some bacterial species, and this would account for a similar rate of reduction in absolute abundance of bacteria as well as ARGs following secondary treatments in WWTPs [71,73]. Secondly, the increase in the relative abundance of SULI after treatment [71,73] may be due to the SULI gene also existing as an eARG, outside the host antimicrobial-resistant bacterial cells [73]. This is further substantiated by the link of SULI to MGE and high rates of HGT [22,23,75]. Furthermore, the association of various ARGs with multiple host ARB during wastewater processing has been documented previously [76,77]. However, with the exception of bacterial culture or utilisation of sophisticated gene sequencing approaches, it is difficult to determine the definitive bacterial host of ARGs in wastewater.

Dynamic shifts in ARG relative abundance across secondary treatment processes in WWTPs have also been demonstrated in other studies. For example, Osińska et al. (2019) noted more than 90% reduction in total ARG load across secondary treatment processes, but they also showed significant increases in the relative abundance of specific ARGs, including: TETM (a gene for tetracycline resistance) and blaTEM (an extended spectrum beta-lactamase gene) identified via PCR methods [15]. Additionally, the levels of free or adsorbed eARGs may not always be significantly affected by secondary treatment processes [78,79,80,81]. Some authors have noted differences in antibiotic resistance profiles between aerobically and anaerobically digested sludge. Anaerobic digesters might reduce ARG abundance and decrease horizontal transfer of ARGs that is facilitated by MGEs including plasmids [6]. Nonetheless, some paradoxical effects have been seen in anaerobic sludge digesters. While some ARGs were undetectable after anaerobic digestion, other unique ARGs appeared to emerge only after this form of treatment, and this suggests that anaerobic digestion may induce or specifically select for some forms of ARGs [6,82]. It is conceivable, therefore, that this conflicting evidence is a direct result of changes in microbial communities and associated ARGs, following anaerobic treatments. This implies that the net effect of anaerobic reactors in the secondary phase of wastewater treatment is dependent on influent quality and its microbial composition, types and concentrations of antibiotics and other chemicals as well as other factors such as the seasonal changes in temperature, humidity, and rainfall. Thus, the impact of anaerobic treatments in the secondary phase of wastewater treatment cannot be generalised from individual case studies in specific plants. The environmental impact of each WWTP as relates to the spread of antimicrobial resistance should be assessed individually.

### 3.3. Tertiary Wastewater Treatment

Tertiary treatment contributes to wastewater disinfection and is used to remove components, which are not reduced by the secondary treatment, such as pathogenic or non-pathogenic microorganisms and associated iARGs, as well as eARGs. The effectiveness of tertiary treatment and its impact on ARGs varies between tertiary wastewater treatment processes. The commonly used tertiary processes include disinfection via ultra-violet radiation, chlorination, ozone treatment, and the use of Membrane-Bioreactors (MBR).

#### 3.3.1. Chlorination Disinfection

The use of chlorination processes as a disinfectant in WWTP has shown varying outcomes on ARB and ARGs. Studies on this form of disinfectant have reported that chlorination either does not significantly reduce ARGs or in some cases favours the growth of ARB and an increase in the relative abundance and conjugative transfer of ARGs between bacteria [35,83]. For example, investigations using culture methods showed that approximately 40% and 80% of erythromycin-resistance and tetracycline-resistance genes could not be removed by chlorination at a dose up to 300 mg Cl_2_ min/L, respectively [84]. Furthermore, a WWTP in northern China reported that ARB identified by culture methods were more resistant to disinfection by chlorination than were total bacteria [61]. Mao et al. (2015) demonstrated that 12 ARGs identified by qPCR and encoding for resistance against tetracyclines (*TETA*, *TETB*, *TETE*, *TETG*, *TETH*, *TETS*, *TETT*, *TETX*), sulfonamides (*SULI*, *SULII*), quinolones (*QNR*B) and macrolides (*ERM*C) were released from the WWTP at higher concentrations in the effluent than the influent following a tertiary chlorine disinfection dose of 5 mg/L for 30 min [61]. Similarly, treatment of wastewater with 8–9 mg/L of chlorine dioxide for 30 min resulted in up to an 8-fold increase in the abundances of iARGs and up to a 4-fold preferential increase in the abundances of eARGs against macrolides (*ERMB*), tetracyclines (*TETA*, *TETB*, *TETC*), sulfonamides (*SULI*, *SULII*, *SULIII*), beta-lactams (*AMPC*), aminoglycosides (*APH*(*2′*)-*LD*), rifampicin (*KATG*) and vancomycin (*VANA*) [85]. Collectively, these studies suggest that disinfection of wastewater with chlorine may enhance environmental pollution with eARGs and iARGs, thereby compounding the spread of antimicrobial resistance. These observations need to be substantiated in large-scale studies covering a wider geographical distribution.

#### 3.3.2. Ultra-Violet Disinfection

Multiple studies have investigated the efficiency of ultra-violet light (UV) to reduce the load of ARGs in WWTPs. At a UV dose of 27 mJ/cm^2^, a 34–75% removal efficiency of ARBs was observed with no obvious reduction in eight ARGs identified by qPCR [78]. From this study, Lee et al. (2017) suggested that although UV disinfection may be appropriate for the treatment and removal of bacterial cells and ARB, it cannot effectively disassemble eARGs, and it may even facilitate the release of iARGs from bacterial cells during lysis and, as a result, create eARGs. This notion is supported by other studies, which have observed a lack of reduction in ARGs when using UV disinfection [45,86]. Using a UV disinfector with a 45% transmittance of 900 kW and light intensity greater than 1 mW/mm^2^, only a slight change in ARG concentrations was observed between the pre- and post-UV disinfected effluent samples via qPCR, with ~0.5–0.7 orders of magnitude for *TET* genes and 0.3 orders of magnitude for *SUL* and *INTI1* genes [45]. In a comprehensive review, however, it was shown that the removal of ARB and ARGs from wastewater depended on several factors, including the nature of bacteria (Gram-positive or Gram-negative), whether targeted ARGs were harbored on plasmids or, chromosomally, whether UV treatment was coupled with other treatments such as advanced oxidation with hydrogen peroxide or chlorination, and the dose and duration of UV treatment [87]. Overall, UV disinfection alone is unlikely to be highly effective in the removal of ARGs from wastewater on a large scale.

#### 3.3.3. Ozone Treatment

Preliminary research on the effectiveness of ozone in reducing ARGs and ARB in wastewater is promising, and this technology may be effective, but studies on this issue are limited, and most have relied on using a mixture of specified bacterial culture and qPCR methods. In laboratory-scale experiments, the intracellular genes of a wastewater bacterial community were disrupted at a specific ozone dose (0.55 g of O_3_ per dissolved organic carbon) [88]. This dose was feasible for full-scale application but had not been applied anywhere, practically, at the time of the initial publication [88]. Another related study demonstrated that at a concentration of 2 milligrams per litre, ozone provided higher removal efficiency of ARGs than UV or chlorination [89]. However, Czekalski et al. (2016) also found that some ARGs within *Escherichia coli* cultures, including the *SULI* gene, were unaffected by ozonation, suggesting that ozone at these doses may not eliminate all ARGs and may be selective in ARG removal [88]. Furthermore, both UV and ozone disinfection may result in cell lysis, with bacterial DNA being released into the environment and the resulting ARGs ending up as free DNA in the treated wastewater [66,89].

#### 3.3.4. Membrane Filtration

The comparably recent implementation of high-efficiency membranes in some WWTPs has significantly reduced microbial concentration in wastewater effluent [90]. Membrane Bioreactor (MBR) plants have several advantages when compared to the conventional use of activated sludge clarification. MBRs have been proven to be effective in removing organic and inorganic matter [16]. MBRs are suggested to be among the most effective removal systems for ARB and their accompanying ARGs [91,92,93,94]. Wang et al. (2020) stated that for some eARGs, including *INT1*, *blaTEM*, *ERMB*, *TETO*, and *TETW*, the average removal through a full-scale MBR process approached 100%, as quantified by qPCR [91]. Similarly, utilising high-throughput qPCR to investigate 319 ARGs and 57 MGEs, a 98.4% decrease in ARGs within an MBR plant was reported by Lin et al. (2021) [92]. However, this study by Lin et al. (2021) also observed that 35 ARGs and 14 MGEs were persistent in all samples, including in the MBR effluent [92]. Furthermore, MGEs in the form of plasmids have been documented to pass through ultra-filtration membrane pores that are smaller than their DNA diameter possibly due to supercoiling [95]. This supercoiling of plasmid DNA is also enhanced by the hydrodynamic pressure caused by transmembrane gradient, and this may be the cause of many eARGs crossing ultra-filtration membrane barriers. Collectively these observations suggest that WWTPs with MBR plants may be effective in the removal of iARGs and/or eARGs, but they are not a perfect system, and effluents from such treatment plants may still contain eARGs at biologically significant concentrations [92]. Further studies on the elucidation of the specific roles of MGEs, iARGs and eARGs in the dissemination of antimicrobial resistance from WWTPs with MBRs are warranted.

## 4. Assessing the Risk of Antibiotic Resistance from WWTPs in the Environment

Many of the currently suggested antimicrobial resistance risk assessment scaffolds have predominantly focussed on public health and include measures of antibiotics, pathogenicity of ARB and their correlations with high risk ARGs [17,46,96,97,98,99,100]. A thorough One Health risk assessment in this field must consider the impact on public health and environmental ecosystems in the context of other factors such as microbial diversity and functions performed by environmental microbiomes, the functionality of ARGs, and effects on sentinel environmental animal or plant species [101,102]. Identifying ARGs and how ARGs spread is crucial to understanding the potential risk for resistance within an environment. However, ARB do ultimately determine the implications of antibiotic resistance either to public health via altered pathogenicity and morbidity or altered microbial diversity and function [103,104,105].

Direct effects of antibiotic use on microbial diversity in humans and animals have been well documented [106,107,108,109,110], with indications of functional effects to the immune system, metabolism, and other important biological functions. Whilst a similar level of functional effect has been hypothesised to contribute to loss of microbial diversity in environmental microbiomes [106,107], very limited information is available on the functional impact of this dysbiosis within natural ecosystems [108]. As such, frameworks on the true functional risks to environmental microbiomes are under-informed. Nonetheless, some progress has been registered in this domain, and the contribution of microbial diversity to environmental effects such as carbon dioxide production, nitrogen fixation, and pH stabilisation have been suggested [108,109,110].

### 4.1. Interactions between ARB, ARGs, MGEs and Co-Occurrence Genes for Risk Scafolding

An appropriate antibiotic resistance risk assessment framework should also incorporate the characterisation of the fate and transport of ARBs, ARGs and MGEs. Many complex interactions lead to HGT and development of antibiotic resistance within specific bacterial cells. One such critical interaction involves co-occurrence genes that describe ARGs that are located on the same MGE or chromosome as genes encoding for other forms of resistance such as metal or biocide resistance [111]. This can create a situation where antibiotic and heavy metal resistance increase in abundance when the afflicted ARB are exposed to either the specified metal, antibiotic, or both stressors [111]. The effect of this specific type of interaction when categorising risk to One Health components is largely unknown. However, accurate genomic approaches can be used to characterise shifts in abundances and diversity of microbial communities and accompanying ARGs; preliminary studies suggest that the combination of ARGs with MGEs plays an important part in the dissemination of antimicrobial resistance in environmental ecosystems [112,113,114,115]. ARGs associated with the co-occurrence of heavy metals or biocide resistance genes have been found to increase in proportion with high abundances of these compounds [112,113,114]. It is conceivable that the co-occurrence of genes related to resistance to other stressors may impact the capability and accuracy of a risk scaffold used to predict the effect of antibiotic resistance pollution within any environmental ecosystem. All these aspects should be included in risk assessments.

### 4.2. A Proposed Pathway to Informed Risk Scaffolds for Antibiotic Resistance from WWTPs

Long-term surveillance of varied WWTPs and their upstream influent sources and downstream receiving environments may provide the insight necessary to make future investment decisions on infrastructure used to manage wastewater based on One Health risk scaffolds. In programs for the systematic surveillance of WWTPs for antimicrobial resistance specifically, risk assessments must be individualised to eliminate bias that may be attributed to quantitative and qualitative differences in influents to WWTPs and differences in wastewater treatment processes. Additionally, analyses of samples from WWTPs and environments into which effluents are discharged should account for the spatial as well as temporal distribution of resistomes. This provides an insight into how wide the antibiotic resistance is spread from sources of pollution and within different matrices, including water, the sediment, flora and fauna. Insight into how this form of pollution may be influenced by seasonal variations or climate change is also provided. The three pillars of One Health (Public Health, Animal Health, and Environmental Health) have been identified as potentially at risk from the indiscriminate dissemination of ARGs [115], with human and animal health threats related to unknown alterations of microbiomes and subsequent dysbiosis, as well as environmental hazards relating to selection pressure and the further rise of environmental antibiotic resistance.

In summary, we provide a structured outline of some of the critical elements required to develop a risk assessment framework for antimicrobial resistance associated with wastewater processing. Herein, we have emphasised the following critical components: (i) accurate identification of the varied sources for antibiotic resistance in wastewater; (ii) characterisation of pathways and fate of the different determinants of antibiotic resistance, including ARB, ARGs, co-occurrence genes, antibiotics and other biocides; (iii) assessing exposure and quantification of risk by determining pathways through which humans, animals and the environment may be exposed and using antimicrobial MICs or probabilistic PNECs as well as changes in abundance or diversity of ARGs and ARB to estimate the likelihood of adverse outcomes; (iv) identifying mitigation strategies by evaluating wastewater treatment processes and technologies. These risk scaffolding components should be used to establish monitoring programs to track incidences of antimicrobial resistance in WWTPs and receiving environments, but it is also critically important to periodically evaluate the effectiveness of any instituted antimicrobial resistance control and monitoring programs.

## Appendix A

**Table A1:** Commonly identified ARGs in WWTPs and methods of detection.

Publication	Study Focus	Treatment in WWTP	Location	ARG Detection Method	ARGs Identified in Influent	Changes to ARG Relative Abundance in Effluent
Chen and Zhang, 2013 [45].	Effect of treatment on ARGs	Activated Sludge	China	qPCR (abundance relative to 16s)	*TETM*, *TETO*, *TETQ*, *TETW*, *SULI*, *SULII*, *INTI1*	*TETM*, *TETO*, *TETQ*, *TETW*, *SULI*, *SULII*, *INTI1*
		constructed wetland		qPCR (abundance relative to 16s)	*TETM*, *TETO*, *TETQ*, *TETW*, *SULI*, *SULII*, *INTI1*	*TETM*, *TETO*, *TETQ*, *TETW*, *SULI*, *SULII*, *INTI1*
		ultraviolet (UV) disinfection		qPCR (abundance relative to 16s)	*TETM*, *TETO*, *TETQ*, *TETW*, *SULI*, *SULII*, *INTI1*	*TETM*, *TETO*, *TETQ*, *TETW*, *SULI*, *SULII*, *INTI1*
Conco, et al, 2022 [35].	ARGs identified in hospital waste	Chlorination	South Africa	WGS	Resistome identified	*SULI*, *AADA4–5*, *TEM-1D* Several ARGs were downregulated
Czekalski, et al, 2012 [31].	Multi-drug resistant bacteria in wastewater stream	Activated Sludge	Switzerland	Bacterial culture, antibiotic sensitivity testing, and qPCR	*SULI*, *SULII*	*SULI*, *SULII*
Czekalski, et al., 2016 [88]	Escherichia coli	ozonation	Germany	Bacterial culture, and qPCR	*SULI*	*SULI*
Gao, et al., 2012 [71].	tetracycline and sulfonamide resistance genes	Activated Sludge	USA	qPCR (abundance relative to 16s)	*TETO*, *TETW*, *SULI*	*SULI*, *TETO*, *TETW*
Ju, et al., 2019 [59].	Resistome	Activated Sludge	Switzerland	WGS (abundance relative to total sequences)	Resistome identified	Several ARGs for multi-drug, beta-lactam, aminoglycoside, tetracycline, trimethoprim, vancomycin, bacitracin, chloramphenicol, rifamycin, sulfonamide, macrolide.
Karkman, et al., 2016 [26].	Resistome	Activated Sludge and biofilters	Finland	qPCR (abundance relative to 16s)	*ERMF*, *blaTEM*, *TETA*, *TNPA*, *ERMB*, *SULII*, *AACC*, *TNPA*, *MEXF*, *TNPA*, *TETO*, *TETW*, *STRB*, *AADA*	*blaTEM*, *TETA*, *TNPA*, *ERMB*
Lee, et al., 2017 [78].	Impact of UV disinfection on ARGs and ARB	ultraviolet (UV) disinfection	China	qPCR (abundance relative to 16s)	*TETX*, *TETM*, *TETA*, *SULI*, *SULII*, *ERMB*, *QNRD*, *blaTEM*	*TETA*, *TETX*, *TETM*, *SULI*, *SULII*, *ERMB*, *QNRD*, *blaTEM*
Li, et al., 2015 [36].	Relationship of Antibiotics to ARGs	ultraviolet (UV) disinfection and constructed wetland	China	qPCR (abundance relative to 16s)	*TETA*, *TETB*, *TETC*, *TETG*, *TETL*, *TETM*, *TETO*, *TETQ*, *TETW*, *TETX*, *SULI*, *SULII*	*TETC*, *TETG*, *TETM*, *TETX*, *SULI*, *INTI1*, *TETA*, *TETB*, *TETL*, *TETO*, *TETQ*, *TETW*, *SULII*
Li, et al., 2018 [14].	pharmaceutical wastewater treatment plant	Activated Sludge	China	qPCR (abundance relative to 16s)	*TETB*, *TETW*, *SULI*, *SULII*, *GYRA*, *QEPA*, *ERMB*, *ERMF*, *INTI1*, *INTI2*	*TETB*, *SULI*, *SULII*, *GYRA*, *INTI1*, *ERMB*, *ERMF*, *QEPA*, *INTI2*
Lin, et al., 2021 [92].	Relationship of Antibiotics to ARGs	Membrane bioreactors	China	qPCR (abundance relative to 16s)	Unspecified 319 ARGs	*APHA3*, *MERA*, *DFRA14*, *APH3-III*, *ERMF*, *ARR-2*, *TET32*, *CEFA*, *blaTEM*, *AACC2*, *DFRA1*, *CN1A5*, *AADA16*, *AADA6*, *AAC(6)-LB*, *COPA*, *MEFA*, *ERMQ*, *INU(F)*, *AADA_99*, *AAC(6)*, *CATB3*, *QACH_351*, *AADA5*, *blaVEB*, *AADA21*, *EREB*, *AADA17*, *MSRE*, *ERMB*, *TETM*, *TETW*, *TETA*, *SULI*, *MPHA*, Expression of all other ARGs was maintained or downregulated
Liu, et al., 2019 [7].	Relationship of Antibiotics to ARGs	ultraviolet (UV) disinfection	China	qPCR (abundance relative to 16s)	*TETA*, *TETC*, *TETQ*, *TETW*, *TETX*, *SULI*, *SULII*, *SULIII*, *INTI 1*	*TETA*, *TETC*, *TETQ*, *TETW*, *TETX*, *SULI*, *SULII*, *SULIII*, *INTI1*
Liu, et al., 2018 [85].	Intracellular and extracellular ARGs	Chlorination	China	qPCR (abundance relative to 16s)	*VANA*, *DFRA1*, *CATA1*, *KATG*, *RPOB1*, *APH(2′)-ID*, *AADA*, *QNRA*, *GYRA*, *AMPC*, *blaTEM*, *ERMB*, *ERMA*, *SULIII*, *SULII*, *SULI*, *TETX*, *TETQ*, *TETM*, *TETC*, *TETB*, *TETA*	*ERMB*, *TETA*, *TETB*, *TETC*, *SULI*, *SULII*, *SULIII*, *AMPC*, *APH(2’)-ID*, *KATG*, *VANA*, *DFRA1*, *CATA1*, *KATG*, *RPOB1*, *AADA*, *QNRA*, *GYRA*, *blaTEM*, *ERMA*, *TETX*, *TETQ*, *TETM*
Mao, et al., 2015 [61].	Relationship of Antibiotics and Heavy metals to ARGs	Chlorination	China	qPCR (abundance relative to 16s)	*TETA*, *TETB*, *TETC*, *TETD*, *TETE*, *TETG*, *TETH*, *TETJ*, *TETK*, *TETL*, *TETM*, *TETO*, *TETQ*, *TETT*, *TETW*, *TETX*, *TETZ*, *SULI*, *SULII*, *SULIII*, *SULA*, *QNRA*, *QNRB*, *QNRD*, *QNRS*, *ERMB*, *ERMC*	*TETA*, *TETB*, *TETE*, *TETG*, *TETH*, *TETS*, *TETT*, *TETX*, *SULI*, *SULII*, *QNRB*, *ERMC*, *TETC*, *TETD*, *TETJ*, *TETK*, *TETL*, *TETM*, *TETO*, *TETQ*, *TETW*, *TETZ*, *SULIII*, *SULA*, *QNRA*, *QNRD*, *QNRS*, *ERMB*
Machado, et al., 2023 [77].	Effect of different treatment types	Activated Sludge	Brazil	Bacterial culture, antibiotic sensitivity testing, and qPCR	*INT1*, *blaTEM*, *TETA*, *SULI*, *QNRB*, *EMRB*	*INT1*, *blaTEM*, *TETA*, *SULI*, *QNRB*, *EMRB*
		anaerobic sludge blanket Reactor				*blaTEM*, *TETA*, *QNRB*, *INT1*, *SULI*, *EMRB*
		ultraviolet (UV) disinfection				*QNRB*, *EMRB*, *INT1*, *blaTEM*, *TETA*, *SULI*
Munir, et al., 2011 [72].	Effect of different treatment types	Activated Sludge	USA	qPCR (abundance relative to 16s)	*TETW*, *TETO*, *SULI*	*TETW*, *TETO*, *SULI*
		Oxidative Ditch				*TETW*, *TETO*, *SULI*
		Membrane bioreactors				*TETW*, *TETO*, *SULI*
Narciso-da-Rocha, et al., 2018 [68].	Effect of treatment on ARGs	ultraviolet (UV) disinfection	Portugal	qPCR (abundance relative to 16s)	*INTI1*, *blaTEM*, *blaOXA-A*, *blaSHV*, *blaCTX-M*, *SULI*, *SULII*, *QNRS*	*blaTEM*, *blaOXA-A*, *QNRS*, *INTI1*, *SULI*, *blaSHV*, *blaCTX-M*, *SULII*
Neudorf, et al., 2017 [32].	Effect of treatment type	constructed wetland	Canada	qPCR (abundance relative to 16s)	*INT1*, *SULI*, *SULII*, *TETO*, *ERMB*, *MECA*, *blaCTX-M*, *blaTEM*, *QNRS*	*SULI*, *SULII*, *MECA*, *TETO*, *QNRS*
Osińska, et al., 2019 [15].	Culture-based identification	Activated Sludge, Plus Anerobic-aerobic reactor	Poland	Bacterial culture, antibiotic sensitivity testing, and qPCR	*blaTEM*, *blaSHV*, *blaOXA*, *TETA*, *TETM*	TETA, TETM, blaTEM, blaSHV, blaOXA
		Plus mechanical & biological system	Poland	Bacterial culture, antibiotic sensitivity testing, and qPCR	*blaTEM*, *blaSHV*, *blaOXA*, *TETA*, *TETM*	*TETM*, *TETA*, *blaTEM*, *blaSHV*, *blaOXA*
		Plus Sequencing batch reactor	Poland	Bacterial culture, antibiotic sensitivity testing, and qPCR	*blaTEM*, *blaSHV*, *blaOXA*, *TETA*, *TETM*	*TETM*, *TETA*, *blaTEM*, *blaSHV*, *blaOXA*
		Plus mechanical & biological nutrient removal	Poland	Bacterial culture, antibiotic sensitivity testing, and qPCR	*blaTEM*, *blaSHV*, *blaOXA*, *TETA*, *TETM*	*TETM*, *TETA*, *blaTEM*, *blaSHV*, *blaOXA*
Pazda, et al., 2020 [79].	Effect of different treatment types on tetracycline and sulfonamide resistance genes	Activated Sludge	Poland	qPCR (abundance relative to 16s)	*TETA*, *TETB*, *TETC*, *TETG*, *TETL*, *TETM*, *TETO*, *TETQ*, *TETX*, *SULI*, *SULII*, *SULIII*	*TETB*, *TETG*, *TETH*, *TETS*, *TETT*, *TETX AND SULI*, *SULII*
		constructed wetland				*TETB*, *TETK*, *TETL*, *TETO*, *SULIII*
Rafraf, et al., 2016 [86].	Effect of different treatment types on resistance genes	Activated Sludge	Tunisia	qPCR (abundance relative to 16s)	*blaCTX-M*, *blaTEM*, *QNRA*, *QNRS*, *SULI*, *ERMB*, *INTI1*	*QNRS*, *ERMB*
		constructed wetland				*blaCTX-M*, *blaTEM*, *AND QNRA*, *INTI1*
Rodriguez-Mozaz, et al., 2015 [73].	hospital-urban wastewater system	Activated Sludge	Spain	qPCR (abundance relative to 16s)	*blaTEM*, *ERMB*, *QNRS*, *SULI*, *TETW*	*blaTEM*, *SULI*, *QNRS*, *ERMB*, *TETW*
Sui, et al., 2019 [80].	Intracellular and extracellular ARGs	Activated Sludge	China	qPCR (abundance relative to 16s)	*TETM*, *TETW*, *TETG*, *TETX*, *ERMB*, *ERMF*, *MEFA*, *EREA*, *SULI*, *SULI*, *blaTEM*, *INTI1*	*MEFA*, *TETM*, *TETW*, *SULI*, *TETG*, *TETX*, *ERMB*, *ERMF*, *EREA*, *SULI*, *blaTEM*, *INTI1*
Wang, et al., 2020 [91].	Intracellular and extracellular ARGs	Membrane bioreactors	China	qPCR (abundance relative to 16s)	*INTI*, *blaTEM*, *ERMB*, *TETO*, *TETW*	*INTI1*, *blaTEM*, *ERMB*, *TETO*, *TETW*
Wen, et al., 2016 [65].	Effect of treatment on ARGs	ultraviolet (UV) disinfection	China	qPCR (abundance relative to 16s)	*TETA*, *TETO*, *TETW*, *SULI*, *SULII*, *blaCTX-M*, *blaTEM*, *blaSHV*, *INTI1*	*TETA*, *TETO*, *TETW*, *SULI*, *SULII*, *blaCTX-M*, *blaTEM*, *blaSHV*, *INTI1*
Xu, et al., 2015 [63].	Relationship of Antibiotics to ARGs	Activated Sludge	China	qPCR (abundance relative to 16s)	*TETA*, *TETB*, *TETE*, *TETW*, *TETM*, *TETZ*, *SULI*, *SULII*, *SULIII*, *GRYA*, *PARC*, *QNRC*, *QNRD*	*QNRC*, *TETM*, *QNRD*, *PARC*, *GYRA*, *TETA*, *TETB*, *TETE*, *TETW*, *TETZ*, *SULI*, *SULII*, *SULIII*
Yang, et al., 2014 [37].	Resistome	Activated Sludge	China	WGS (abundance relative to total sequences)	Resistome identified	ARGs for Sulfonamide, Quinolone and Chloramphenicol resistance. Beta-lactams, Tetracyclines, Aminoglycosides
Yuan, et al., 2019 [81].	Intracellular and extracellular ARGs	Activated Sludge	China	qPCR (abundance relative to 16s)	*TETA*, *TETC*, *TETM*, *TETX*, *SULI*, *SULII*, *blaTEM*, *EREA*, *ERMB*	*TETA*, *TETC*, *TETM*, *TETX*, *SULI*, *SULII*, *blaTEM*, *EREA*, *ERMB*

ARGs highlighted in green were identified as having significantly increased in relative abundance in the effluent of WWTPs compared to the relative abundance in the influent. ARGs highlighted in red were observed to either significantly decrease or not change in relative abundance in the effluent compared to the relative abundance in the influent.
